# Dynamic Performance Evaluation of Optical and Capacitive Sensors for Atmospheric Water Vapor Monitoring

**DOI:** 10.3390/s26134064

**Published:** 2026-06-26

**Authors:** Mattia S. Arrigo, Roberto M. R. Di Martino, Marcello Liotta

**Affiliations:** 1 Dipartimento di Scienze della Terra e del Mare, Università degli Studi di Palermo, 90133 Palermo, Italy; 2 Istituto Nazionale di Geofisica e Vulcanologia, Sezione di Palermo, 90146 Palermo, Italy; roberto.dimartino@ingv.it

**Keywords:** water vapor, spectroscopy, capacitive sensors, greenhouse

## Abstract

Atmospheric water vapor (H_2_O) is the most significant greenhouse gas; accurately quantifying it is a critical yet challenging task due to its high spatial and temporal variability. This study presents a comparative analysis of the LI-COR LI-7825 (LI-COR Biosciences, Lincoln, Nebraska), which utilizes OF-CEAS technology, and the Vaisala HMP7 (Vaisala Oyj, Vantaa, Finland) capacitive probe, which employs HUMICAP ^®^ technology. Before testing the sensors under dynamic conditions, we performed a two-point calibration on the Vaisala HMP7. We then proceeded to evaluate the two technologies using a custom-made setup to put them in series and minimize atmospheric contamination. The results demonstrate a high statistical correlation $\left(R^{2}=0.995\right)$, though the Vaisala sensor exhibited systematic offsets at high-humidity levels. To better compare datasets, a low-pass digital filter was used to account for the different T_90_ values of the instruments. The results show that while spectroscopy provides superior precision and response speed, the environmental resilience of capacitive sensors makes them better suited for field work. The implementation of robust synchronization protocols is essential to develop effective hybrid monitoring networks that balance precision with scalability.

## 1. Introduction

It is widely known that water vapor (H_2_O) represents the most significant greenhouse gas in Earth’s atmosphere, exerting a radiative forcing that far exceeds that of carbon dioxide (CO_2_) or methane (CH_4_) [1].

The amount of water vapor contained in a volume of gas mixture is primarily driven by temperature via the Clausius–Clapeyron relation [2,3]; therefore, H_2_O acts as a strong internal feedback mechanism that amplifies the warming effect initiated by anthropogenic CO_2_ emissions [1]. This “water vapor feedback” is a critical component of climate sensitivity; as the planet warms, the atmosphere’s capacity to hold moisture increases, further trapping longwave radiation and creating a self-reinforcing thermal cycle [4].

The interaction between H_2_O and CO_2_ is not merely additive but synergistic [5]. Anthropogenic perturbations in the carbon cycle drive an initial temperature rise, which subsequently increases evaporation and atmospheric humidity. This shift not only alters the global energy budget but also modifies precipitation patterns, cloud formation, and the frequency of extreme meteorological events [6]. Monitoring these fluctuations with high temporal and spatial resolution is therefore essential for validating climate models and improving our predictive capabilities regarding the greenhouse effects. However, accurately quantifying atmospheric water vapor remains a significant technical challenge due to its high spatial variability and the wide dynamic range of concentrations in the atmosphere.

Traditionally, capacitive sensors have been employed due to their robustness [7]. The demand for higher precision and faster response times has driven significant advancements in both capacitive materials [8] and laser-based spectroscopic systems. However, a key operational difference remains in practical field applications, where researchers must constantly choose between the absolute precision of sophisticated optical layouts and the immediate, user-friendly scalability of commercially ruggedized probes. For a long time, the optical sensor technologies remained confined to controlled laboratory settings due to their complexity, power requirements, and size, until the emergence of a new generation of field-deployable instruments, which finally bridged the gap between high-precision lab analysis and on-site applications [9,10].

This study provides a comparative analysis of two different instruments: the LI-COR LI-7825, which utilizes Optical Feedback Cavity Enhanced Absorption Spectroscopy (OF-CEAS) for high-precision isotopic (δ^13^C-CO_2_, δ^18^O-CO_2_, and δ^17^O-CO_2_) and concentration measurements (H_2_O, CO_2_, and ammonia—NH_3_), and the Vaisala HMP7, a robust probe based on HUMICAP^®^ capacitive technology designed for high-stability monitoring in demanding environments. By evaluating their performance, we aim to define the operational limits and specific advantages of spectroscopy versus capacitive sensing to find the balance between high temporal/spatial resolution and field scalability in the context of modern atmospheric research.

## 2. Materials and Methods

### 2.1. Technical Details of LI-COR LI-7825 and Vaisala HMP7

The LI-COR LI-7825 (LI-COR Biosciences, Lincoln, Nebraska) sensor technology relies on OF-CEAS technology. Unlike traditional spectrometers that pass a beam through a sample once, LI-7825 utilizes an optical cavity composed of two reflective mirrors facing each other which multiply the path length of the laser beam in the measurement cell.

The “Optical Feedback” involves a portion of the laser light reflecting from the cavity into the laser diode, which forces the laser to lock its frequency to the cavity’s resonance. This results in a beam that bounces back and forth thousands of times, creating an effective optical path length of several kilometers within a physical space only a few centimeters long. As the air sample is drawn into the cavity, water molecules absorb light at a very specific near-infrared frequency. By measuring the attenuation of the light, the instrument calculates a near-instantaneous concentration. Because it targets a very specific near-infrared frequency (~1963 nm), corresponding to a roto-vibrational energy level of the water molecule, it is virtually immune to interference from other gases. Further technical specifications are available in the comprehensive datasheet provided by the manufacturer [11].

The Vaisala HMP7 (Vaisala Oyj, Vantaa, Finland) takes a more mechanical approach to moisture. It utilizes the proprietary HUMICAP^®^ (Vaisala Oyj, Vantaa, Finland) sensor, which is essentially a thin-film capacitor. The dielectric layer is a specialized polymer that absorbs and desorbs water molecules in proportion to the ambient relative humidity. As water molecules enter the polymer, they change its dielectric constant, which in turn shifts the capacitance of the sensor. The HMP7’s electronics measure this capacitance change and convert it into a humidity value. An important feature is its integrated heating element, which prevents water condensation on surfaces and ensures outstanding performance in high-humidity environments, where other capacitive sensors usually achieve saturation. Further technical specifications are available in the comprehensive datasheet provided by the manufacturer [12].

### 2.2. Water Vapor Saturated Mixture Preparation

**Figure 1:**
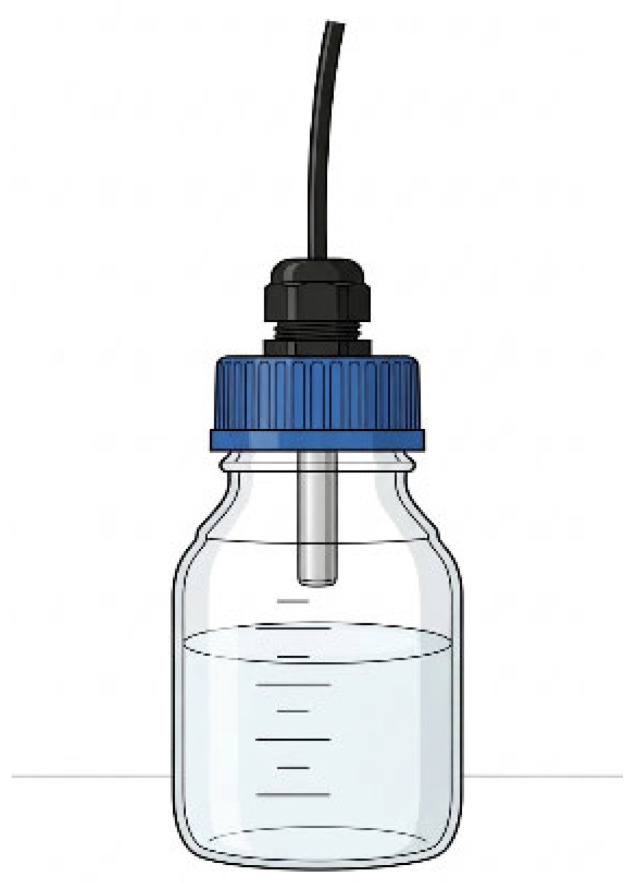
System used to perform Vaisala calibration. We ensured no atmospheric contamination and no liquid touching probe. EPS container not shown here.

For the calibration of the Vaisala HMP7 probe we followed a standard protocol using water vapor saturated aqueous solutions. Magnesium dichloride (MgCl_2_) and sodium chloride (NaCl) have been used to create controlled-humidity environments. Figure 1 depicts the setup we used to calibrate the probe; we prepared the salted solutions in a Simax 250 mL glass bottle [13] with a GL45 cap. In the closed liquid vapor system, at a given temperature, the water vapor saturation in the headspace depends on the salt solubility in the liquid phase. The solubility values for NaCl and MgCl_2_ were provided by IUPAC [14]. The probe was inserted through a PG 13.5 cable gland mounted on top of the cap, which was tightened around the cable to ensure a tight seal and prevent any leaks. The system was then thermally insulated in an Expanded Polystyrene (EPS) container where the temperature remained constant at ≈25.5 °C. We then compared our readings against the thermodynamic tables provided by Greenspan [15].

**Figure 2:**
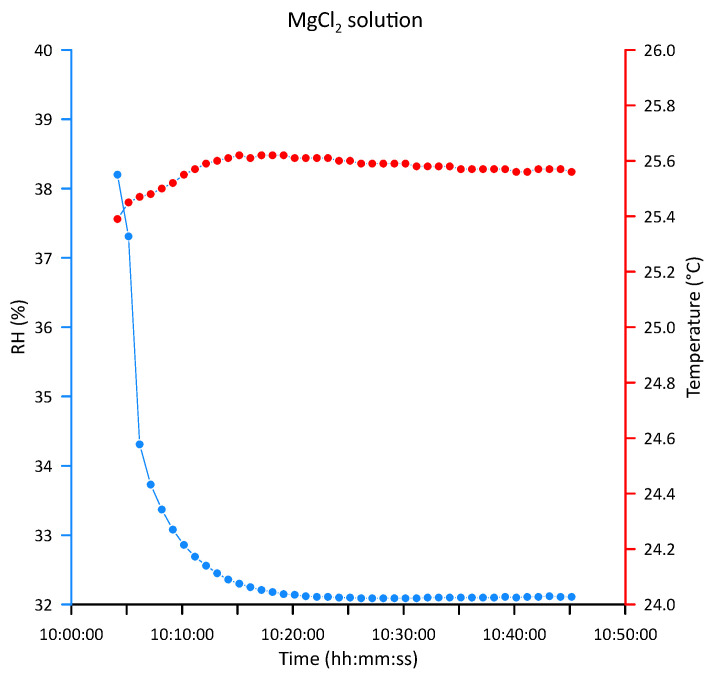
Measured RH (blue) for the magnesium chloride solution and temperature (red) versus time.

Figure 2 shows the experimental curve of the measured relative humidity (RH) as a function of temperature for the saturated magnesium chloride (MgCl_2_) solution; the starting point was the surrounding atmosphere. After a few minutes the measured RH achieves a constant trend.

**Figure 3:**
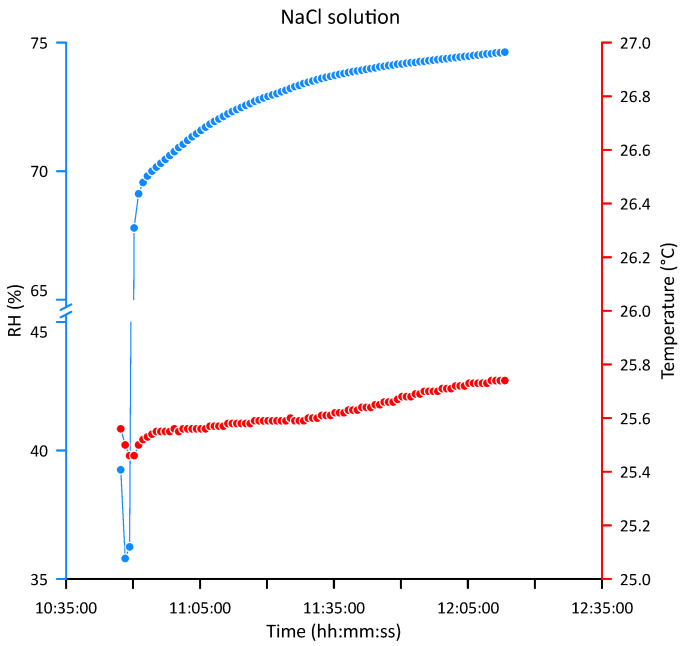
Measured RH (blue) for the sodium chloride solution and temperature (red) versus time.

Figure 3 shows the experimental curve of the measured relative humidity (RH) as a function of temperature for the saturated NaCl solution; the starting point was the surrounding atmosphere.

**Figure 4:**
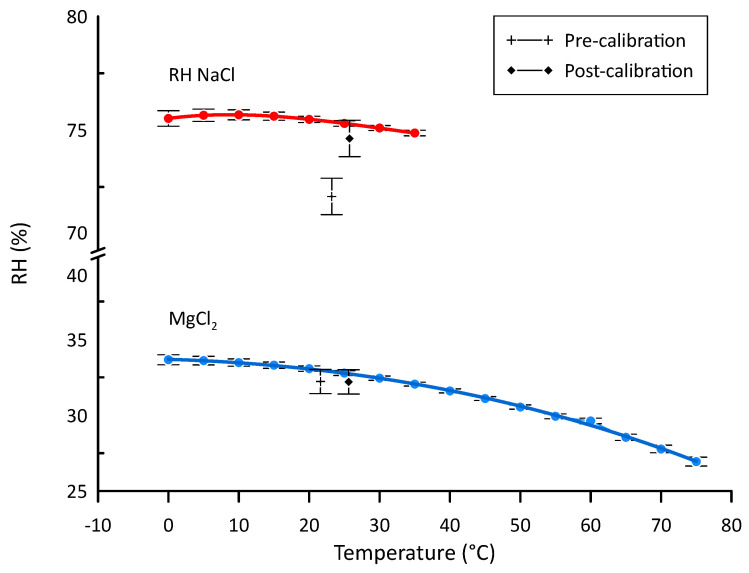
Greenspan’s theoretical values for RH versus temperature for magnesium chloride (blue) and sodium chloride (red). Measured values are depicted using the cross symbol (✛) for pre-calibration data and the diamond (♦) symbol for post-calibration.

Figure 4 illustrates the theoretical trend of RH versus temperature, together with its uncertainty, for both MgCl_2_ and NaCl, in agreement with [15]. The figure also displays the experimental values obtained considering two points pre-calibration and two points post-calibration, along with the measurement uncertainty specified by the manufacturer.

The measured values pre-calibration, mostly for NaCl, deviated from the trends documented in the literature [15]; we therefore decided to perform a two-point calibration. Subsequently, we re-measured the trends for both solutions. After the two-point calibration procedure, consisting of immersing the probe in the saturated solution, waiting for the signal to reach stability, and then entering the known RH value, the measured RH overlapped with the expected values, considering the respective uncertainties.

### 2.3. Dynamic Measurement Setup

**Figure 5:**
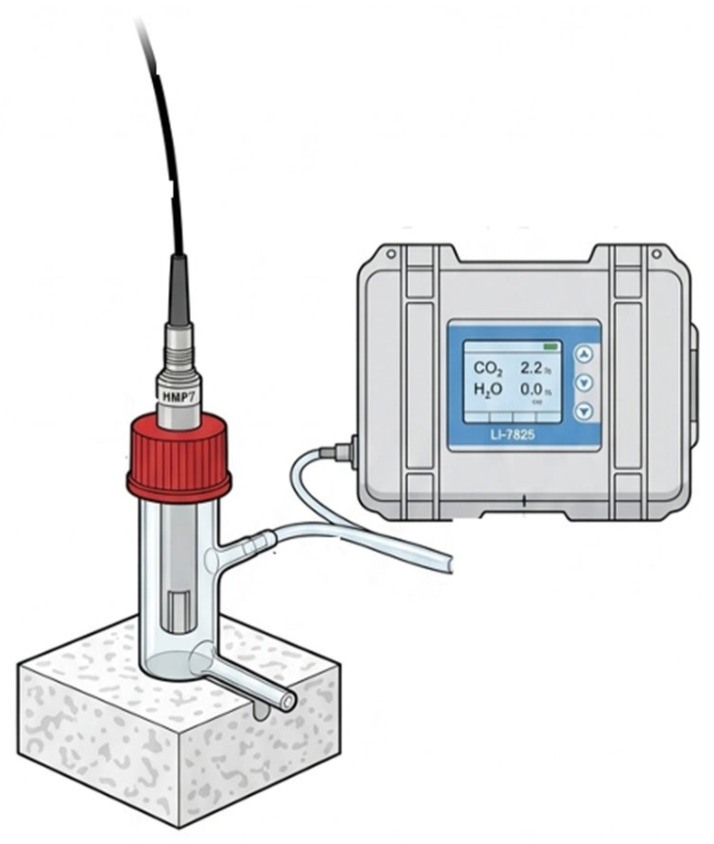
Experimental setup used to put in series the two sensors. Note that the LI-COR display is not positioned at the top in the actual configuration, and the glass stubs are 180° apart, not 90°. The silicone tube is represented as one segment, not three different segments.

After the calibration we proceeded to evaluate the response of the Vaisala HMP7 sensor under dynamic conditions, using a custom glass sampling cell allowing the connection of multiple sensors. This component allowed parallel measurement with the LI-7825 sensor. The cell has a height of 95.20 mm and an inner diameter (i.d.) of 17.46 mm (Figure 5). It features two glass stubs, each 38.57 mm long with an i.d. of 2.87 mm.

The inlet stub is open to the atmosphere via a 55 mm silicone tube with i.d. = 6 mm The outlet stub is connected to the inlet of the LI- 7825 analyzer through a series of three silicone tube segments: a first segment of 70mm length and 6 mm i.d., a second segment of 110 mm length and 6.35 mm i.d., and a final segment of 60 mm length and 6 mm i.d. A schematic depiction is provided in Figure 5, illustrating how the instruments are connected in series to facilitate the visualization of the system’s setup.

The setup was dictated by the logistical availability of specific connectors. It should be noted that the experimental results are independent of tubing dimensions, ensuring full reproducibility.

The total “empty” volume of this glass and silicone assembly was calculated to be 32.01 cm^3^, based on standard cylinder volume geometry ($V=\pi r^{2}h$) for each segment. During the experiment, a Vaisala HMP7 probe was inserted directly into the glass cell. To account for the dead volume occupied by the probe body, its capacity was estimated at approximately 10.74 cm^3^. This yields a net effective gas volume of approximately 21.27 cm^3^ for the entire system up to the LI-COR inlet.

The LI-COR LI-7825 operates at a continuous nominal flow of 250 cm^3^/min (4.16 cm^3^/s).

Dividing the net system volume by this flow rate yields a total theoretical gas residence time of roughly 5.1 s from the atmospheric inlet to the LI-COR optical cell.

Because the Vaisala HMP7 probe is positioned inside the glass cell while the LI-COR is at the end of the sampling line, a physical transport delay occurs between the two measurements. The volume of the line downstream of the glass cell (including the outlet glass protrusion and the three silicone segments) is approximately 7.16 cm^3^. At a flow rate of 250 cm^3^/min, it takes about 2 s for an air parcel sampled by the Vaisala probe to reach the LI-COR inlet.

In addition to the physical transport lag, the inherent response times of the two sensors differ significantly and must be considered for proper data evaluation.

The LI-COR LI-7825 utilizes laser-based spectroscopy with a T_90_ response time of <2 s, while the Vaisala HMP7 utilizes a thin-film polymer capacitive sensor, which relies on chemical diffusion and yields a typical T_90_ response time of approximately 15 s.

Consequently, if a step in change in gas concentration is introduced into the atmospheric inlet at a given time T_0_, the air parcel fills the glass cell at T_0_ + 1.5 s, where the Vaisala probe begins its 15 s ramp toward the new value, and the same air parcel reaches the LI-COR inlet at T_0_ + 3.5 s. Accounting for the analyzer’s 2 s response time, the LI-COR reaches a stable, accurate reading at T_0_ + 5.5 s. Meanwhile, the Vaisala probe does not reach a stable reading of that same air parcel until approximately T_0_ + 16.5 s.

To properly correlate the data from both instruments in the analysis, time-series logs need to be corrected by aligning and accounting for both dead volume and response times.

To prevent any leaks, the borosilicate cell was closed with a GL25 cap [16] and a silicone sealing ring [17], ensuring a closed system and no contamination from the atmosphere.

The experimental procedure was planned to evaluate the response time and the measurement accuracy of the two sensors across different humidity levels. All gas mixtures were prepared in a 10 L Tedlar bag connected to the instruments via the silicone tube joined to the inlet glass stub. This material was specifically selected for its high chemical inertness and extremely low gas permeability, ensuring that the H_2_O concentration remained stable by preventing molecular diffusion through the bag walls and protecting the sample from external atmospheric influence [18].

To achieve water vapor concentrations lower than those of the ambient atmosphere, a mixture was prepared by blending synthetic air (grade 5.5) [19] with ambient air. This setup allowed for a stable reduction in H_2_O ppm_v_ levels. The testing sequence consisted of two distinct pulses: a short-duration pulse of 1 min and a long-duration pulse of 5 min. The pulses were separated by a 3 min recovery interval to observe the sensor’s return to baseline.

**Figure 6:**
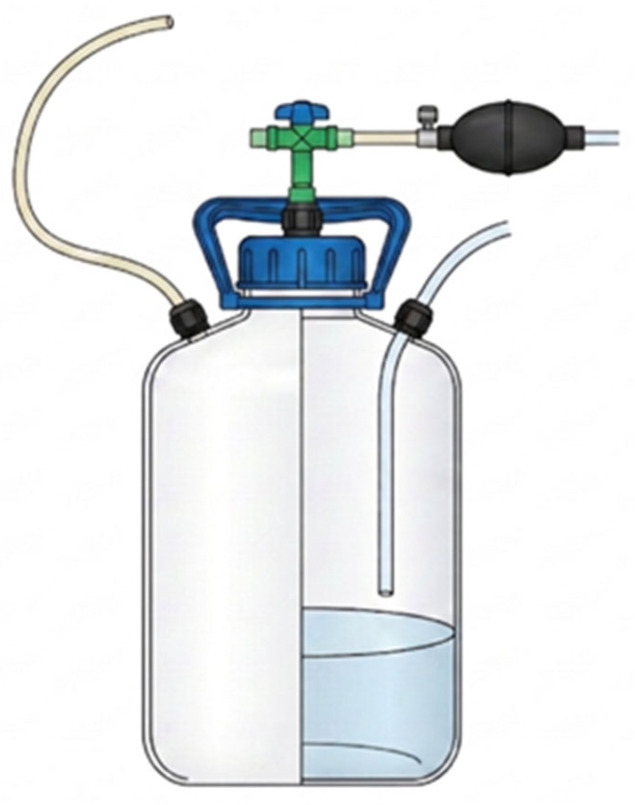
System used to generate air with 100% RH; the air was pumped from the container to the Tedlar bag using a manual pump. Tubes are for letting ambient air reach the system from the bottom.

For H_2_O concentrations exceeding ambient levels (~13,000 ppm on average), a high-humidity source was generated using a closed container with distilled water to create a saturated atmosphere with 100% RH (Figure 6). This saturated air was then mixed with ambient air inside the Tedlar bag to reach the target ppm_v_ values. Consistent with the negative-pulse procedure, the positive pulses were applied in two stages: a 1 min pulse and a 5 min pulse separated by a 3 min recovery time.

## 3. Results

To evaluate the agreement between measurements performed with the two instruments, the H_2_O concentration (ppmv) was monitored across a range of humidity levels (Supplementary Material). Given the different sampling characteristics, specifically the Vaisala’s 5 s acquisition interval, a synchronization protocol was implemented to ensure a meaningful comparison. The LI-COR data were processed using a centered moving average over a 5 s window, for which the LI-COR error drops to 5 ppm. For every Vaisala data point recorded at time t, the corresponding LI-COR value was calculated as the average of all samples within the interval [t − 2 s, t + 2 s]. Because the LI-COR LI-7825 natively reports absolute water vapor volume fractions in ppm while the Vaisala HMP7 natively records relative humidity (RH), a thermodynamic conversion protocol was applied to cross-examine the datasets on an identical absolute molecular scale. The Vaisala RH readings were converted to ppm using the following formula:

(1) $$ppm=\left(\frac{AH\;\times T\;\times R}{\;{MW}_{H2O}\;\times P}\right)\times 10^{6}$$

where AH = absolute humidity (g/m^3^), T = temperature (K), R = 8.3145 (J/mol × K), MW_H2O_ = 18.0154 (g/mol) and *p* = 101,325 (Pa).

**Figure 7:**
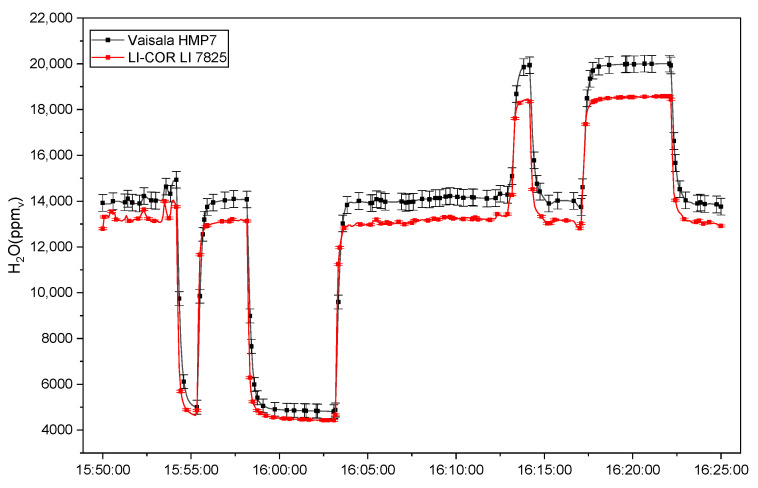
H_2_O ppmv readings from the Vaisala HMP7 (black) compared to the LI-COR LI-7825 (red); error bars reflect the baseline precision specified in the manufacturer’s datasheets.

As illustrated in Figure 7, both sensors exhibit a high degree of correlation in identifying stable humidity plateaus. However, although the fine temporal details are difficult to discern because the figure spans the entire continuous experimental run, systematic offsets remain visible: the Vaisala HMP7 consistently reports slightly higher absolute values during high-humidity phases compared to the LI-COR LI-7825. This systematic divergence is more clearly resolved and can be better appreciated in the localized expansions presented in the subsequent figures.

It is noticeable that the Vaisala HMP7 measurements exhibit strong agreement with the LI-7825 at sub-atmospheric humidity levels (~13,000 ppm on average). Conversely, significant divergence is observed at concentrations exceeding typical atmospheric humidity values.

**Figure 8:**
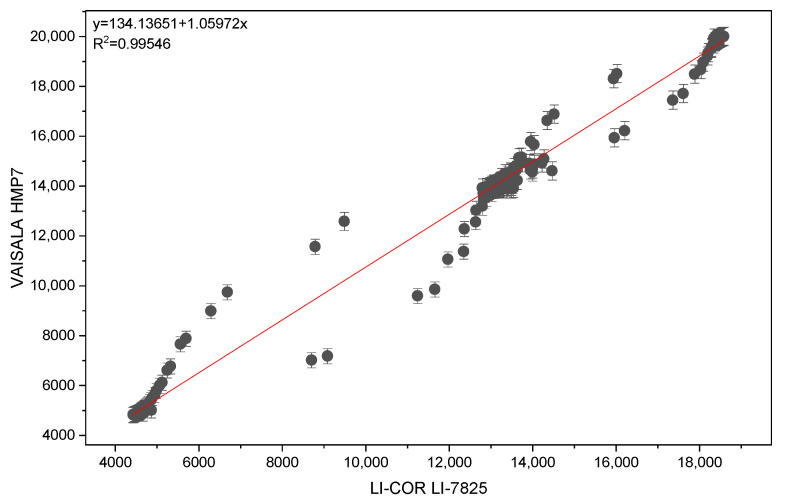
Correlation between Vaisala HMP7 and LI-COR LI-7825 humidity measurements. The red line represents the linear regression fit. Error bars indicate the measurement uncertainty for each data point.

Furthermore, the correlation between the two sensors was statistically evaluated by a linear regression analysis (Figure 8), where the Vaisala HMP7 readings were plotted against the synchronized LICOR LI-7825 moving average.

The linear regression yields a slope of 1.0597 and an intercept of 134.14 ppm_v_, with a coefficient of determination ($R^{2}$) of 0.995. The near-unity slope demonstrates a consistent response between the two instruments across the entire sampled range. However, the intercept and the slight deviation from the ideal 1:1 ratio account for the systematic offsets previously observed, particularly at higher humidity concentrations.

**Figure 9:**
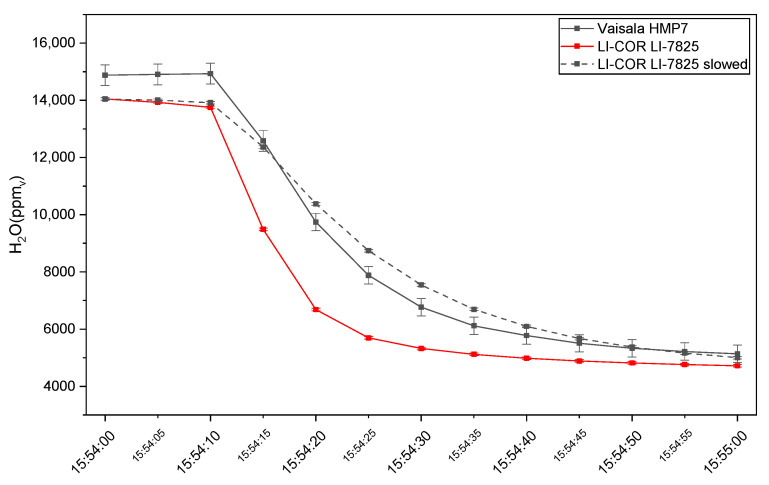
Response of the Vaisala (black) and LI-COR (red) sensors during a sharp increase in humidity levels. The dashed line represents the LI-COR signal slowed to emulate the response time of the Vaisala. Error bars reflect the baseline precision specified in the manufacturer’s datasheets.

**Figure 10:**
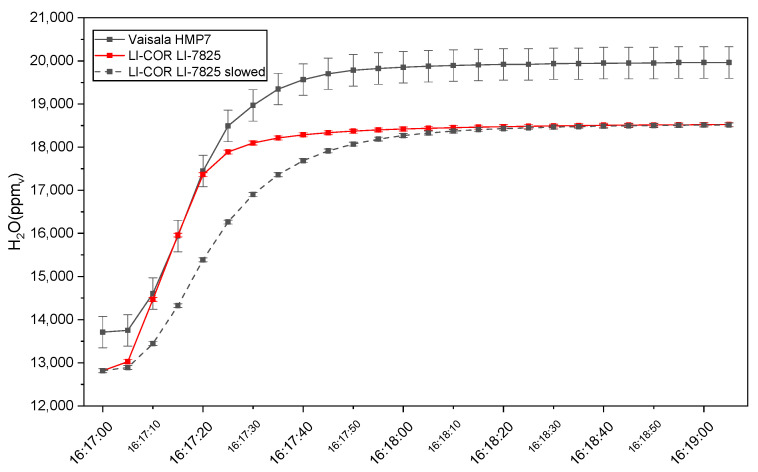
Response of the Vaisala (black) and LI-COR (red) sensors during a sharp decrease in humidity levels. The dashed line represents the LI-COR signal slowed to emulate the response time of the Vaisala. Error bars reflect the baseline precision specified in the manufacturer’s datasheets.

We then proceeded to analyze the dynamic performance of both sensors during sharp humidity levels transitions, depicted in Figure 9 and Figure 10. In both scenarios, during deficit and excess pulses of H_2_O, a clear difference in response times is observed between the two sensors. While the LI-7825 responds almost instantaneously to the gas mixture change, the Vaisala HMP7 exhibits a characteristic damping effect, typical of capacitive sensors [7]. To quantify the response lag of the Vaisala sensor relative to the LI-7825 reference, a first-order low-pass digital filter [20,21] was applied to the LI-7825 signal. This “slowed down” LI-7825 curve was generated to emulate the physical time constant of the Vaisala probe.

The transformation follows the discrete recursive formula [22]

(2) y[n]=y[n−1]+α(x[n]−y[n−1])

where y[n] is the filtered output at the current step, y[n−1] is the previous filtered value, x[n] is the raw LI-COR input signal, and α is the smoothing factor, determined to be 0.35 for this setup.

By applying this filter (Equation (1)), the LI-7825 curve closely mirrors the shape of the Vaisala HMP7 curve during both the rising and falling phases of the pulses.

## 4. Discussion

The comparative analysis presented in this study highlights the critical importance of cross-validating sensors with differing physical principles, especially for measurements in the atmospheric environment. High-precision spectroscopy can be used to “anchor” more accessible field probes, which, beyond being lower-cost sensors, offer significantly greater environmental resilience. The results of this study demonstrate that while cavity-enhanced spectroscopy provides unparalleled precision and near-instantaneous response speed, commercial capacitive field probes offer distinct logistical and structural advantages for long-term outdoor deployment. Their environmental resilience against dust, mechanical vibration, and condensation makes them uniquely suited for scalable field work where the complex operational requirements and alignment vulnerabilities of optical spectrometers would be prohibitive. Therefore, capacitive sensors are not inherently superior, but rather serve as a more pragmatically scalable node in comprehensive monitoring networks when paired with a spectroscopic reference.

Our findings suggest strong agreement between the measurement performed with LI-7825 and Vaisala HMP7 sensors at sub-atmospheric concentration levels; this validates the cooperation between different technologies and the need for comparative analysis to better understand the operational effectiveness of the sensors.

A key finding of this study is that the raw comparison of high-frequency instruments with slower response probes is fundamentally flawed without a robust synchronization protocol. Our study highlights that accounting for both physical transport delays and electronic/chemical lag is not only an appropriate data-processing step, but a physical requirement for meaningful correlation. For researchers managing multi-platform networks, such as combining stationary spectroscopic towers with mobile capacitive sensor nodes, implementing recursive smoothing algorithms is essential to harmonize datasets and avoid systematic errors.

Looking forward, these comparative methodologies pave the way for more sophisticated hybrid monitoring networks. By deploying a limited number of high-precision spectroscopic analyzers as calibration hubs, large-scale arrays of capacitive sensors can be dynamically corrected in the field. This approach is particularly relevant in many fields other than climate monitoring. For example, cost-effective sensors can be an effective solution in agriculture, where localized humidity gradients drive crop health; in industrial process control, where rapid transitions in gas mixtures must be monitored, without the prohibitive cost of deploying multiple spectrometers; and in many other applications in various fields.

The future of atmospheric humidity monitoring lies in the synergy between precision and scalability. As shown in this study, understanding the transfer function between a fast response methodology and a robust field probe allows for a unified data stream that preserves both the accuracy of spectroscopy and the spatial versatility of capacitive sensing.

## Data Availability

The dataset generated and analyzed during the current study are available in the Supplementary Materials file entitled Excel S1: Vaisala_Licor_12_03.xlsx.

## Supplementary Materials

The following supporting information can be downloaded at: https://www.mdpi.com/article/10.3390/s26134064/s1, Excel S1 file regarding the comparative analysis performed on 12 March.
